# Graphene Nanocomposites

**DOI:** 10.3390/molecules24132440

**Published:** 2019-07-03

**Authors:** Xin Zhao, Mo Yang

**Affiliations:** Department of Biomedical Engineering, the Hong Kong Polytechnic University, Hung Hom, Kowloon, Hong Kong, China

Integrating graphene with other nanomaterials has created a variety of graphene nanocomposites with extraordinary chemical, optical, mechanical, and electrical properties. These composites could combine the characteristics of their components to achieve structural stability and multifunctionality with proper design of the interfacial interactions. The field of graphene nanocomposites has developed rapidly over the past decade, with many applications in various fields, such as chemical/biomedical sensing, tissue engineering, and bioimaging.

This Special Issue contains six research articles and two reviews in total, covering structure-proprieties of graphene and its applications in sensing organic solvents, light and DNA, as well as in tissue engineering. Among these, four articles reported graphene nanocomposites for chemical/biomedical sensing applications. One article reported fabrication of graphene-like ZnO/graphene oxide (GO) nanosheets for acetone vapor sensing [1]. The sensing performances of such nanocomposites are superior: the response value could be up to 94 to 100 ppm and the recovery time could be as short as 4 s. Another paper developed a highly-sensitive-plasmonic-resonating sensor consisting of two graphene-modified straight waveguides, two metallic layers, and a racetrack nanodisk resonator [2]. The authors found that their product had the impressive Q value and refractive sensitivity of 21.5 and 1666.67 nm RIU^−1^, respectively. Moreover, this sensor achieved a temperature sensitivity of 2.33 nm/5 °C. These findings can expand the range of use for a nanoscale real-time temperature sensor with resistance to electromagnetic interference. Another project fabricated a temperature sensor consisting of two metal layers and two ethanol-sealed elliptical resonators, connected to a straight waveguide using two rectangular tubes [3]. This sensor can detect temperatures as low as −3.64 nm/°C, far more sensitive than traditional temperature sensors. The fourth paper presented a p-n-p monolayer graphene photodetector doped with TiO_2_ for light detection (405 to 1310 nm) [4].

In one of the remaining two research articles, the authors fabricated chitosan/graphene oxide (CS/GO) composite scaffolds for bone regeneration [5]. The other article describes the structure-properties of GO [6]. In this study, the authors investigated the wettability of a defective GO film using molecular dynamics simulations. They found that the contact angle of GO increased from 70° to 82°, with the increasing defective concentration from 0% to 10%. This finding could give insight on how to control the wetting properties of GO and design new devices. 

One of the two review articles investigates the application of graphene nanocomposites for DNA detection. This review comprehensively summarizes recent trends in DNA detection using graphene and graphene-related nanomaterials including graphene nanopore (GNP), graphene nanoribbon (GNR), GO, reduced graphene oxide (rGO), and graphene-nanoparticle (G-NP) hybrid materials [7]. It also identifies the current challenges and future research directions. For example, the authors mention that although the G-NP hybrids are promising DNA biosensors, improvements are required for the technology to synthesize low-toxicity graphene nanocomposites with controllable size, shape, crystallinity, and defects at a low cost with a high yield. In addition, they suggest synthesizing graphene nanogaps and GNRs to realize the DNA base discrimination. The other review discusses the effect of graphene nanocomposites on neural stem cell differentiation and their applications in neural tissue engineering [8]. The authors conclude with the potential problems to be considered when using graphene nanocomposites including nanoparticle toxicity, aggregation, and intracellular/extracellular reactive oxygen species (ROS) generated during the accumulation of graphene-based materials. This special issue is accessible through the following link: 

https://www.mdpi.com/journal/molecules/special_issues/graphene_nanocomposites
